# 3-Chloro-*N*-(diphenyl­carbamothio­yl)benzamide

**DOI:** 10.1107/S1600536809001639

**Published:** 2009-01-17

**Authors:** Gün Binzet, Ulrich Flörke, Nevzat Külcü, Hakan Arslan

**Affiliations:** a Department of Chemistry, Faculty of Arts and Science, Mersin University, Mersin, TR 33343, Turkey; b Department of Chemistry, University of Paderborn, Paderborn 33098, Germany; c Department of Natural Sciences, Fayetteville State University, Fayetteville, NC 28301, USA; d Department of Chemistry, Faculty of Pharmacy, Mersin University, Mersin, TR 33169, Turkey

## Abstract

In the title compound, C_20_H_15_ClN_2_OS, the bond lengths and angles in the thio­urea group are typical of thio­urea derivatives. The C—N bond lengths in the center of the mol­ecule are shorter than the normal C—N single bond due to the resonance effects in this part of the mol­ecule. The conformation of the title mol­ecule with respect to the thio­carbonyl and carbonyl groups is twisted, as reflected by the C—N—C—O and C—N—C—N torsion angles of −4.4 (6) and −53.3 (5)°, respectively. Pairs of the mol­ecules are linked into centrosymmetric dimers, stacked along the *c* axis *via* inter­molecular N—H⋯S inter­actions. There are also weak inter­molecular C—H⋯O and C—H⋯S contacts in the structure.

## Related literature

For synthesis, see: Özer *et al.* (2009 ▶); Mansuroğlu *et al.* (2008 ▶); Uğur *et al.* (2006 ▶); Arslan *et al.* (2003*c* ▶). For general background, see: Koch (2001 ▶); Huebhr *et al.* (1953 ▶); Madan *et al.* (1991 ▶); Schroeder (1955 ▶). For related structures, see: Khawar Rauf *et al.* (2006 ▶, 2009 ▶); Arslan *et al.* (2003*a* ▶,*b* ▶); Yamin & Yusof (2003 ▶).
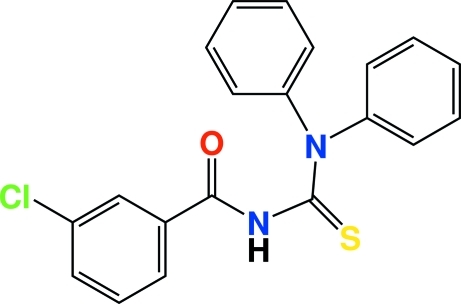


## Experimental

### 

#### Crystal data

C_20_H_15_ClN_2_OS
*M* *_r_* = 366.85
Triclinic, 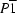

*a* = 8.196 (5) Å
*b* = 10.357 (6) Å
*c* = 11.699 (6) Å
α = 72.565 (10)°
β = 70.495 (10)°
γ = 71.303 (10)°
*V* = 865.8 (9) Å^3^
*Z* = 2
Mo *K*α radiation
μ = 0.35 mm^−1^
*T* = 120 (2) K
0.49 × 0.32 × 0.10 mm

#### Data collection

Bruker SMART APEX diffractometer
Absorption correction: multi-scan (*SADABS*; Bruker, 2002 ▶) *T*_min_ = 0.847, *T*_max_ = 0.966
5401 measured reflections
3490 independent reflections
2290 reflections with *I* > 2σ(*I*)
*R*_int_ = 0.080

#### Refinement

*R*[*F* ^2^ > 2σ(*F* ^2^)] = 0.061
*wR*(*F* ^2^) = 0.146
*S* = 1.06
3490 reflections
230 parameters
1 restraint
H atoms treated by a mixture of independent and constrained refinement
Δρ_max_ = 0.37 e Å^−3^
Δρ_min_ = −0.31 e Å^−3^

### 

Data collection: *SMART* (Bruker, 2002 ▶); cell refinement: *SAINT* (Bruker, 2002 ▶); data reduction: *SAINT*; program(s) used to solve structure: *SHELXTL* (Sheldrick, 2008 ▶); program(s) used to refine structure: *SHELXTL*; molecular graphics: *SHELXTL*; software used to prepare material for publication: *SHELXTL*.

## Supplementary Material

Crystal structure: contains datablocks I, global. DOI: 10.1107/S1600536809001639/bq2121sup1.cif

Structure factors: contains datablocks I. DOI: 10.1107/S1600536809001639/bq2121Isup2.hkl

Additional supplementary materials:  crystallographic information; 3D view; checkCIF report

## Figures and Tables

**Table 1:** Hydrogen-bond geometry (Å, °)

*D*—H⋯*A*	*D*—H	H⋯*A*	*D*⋯*A*	*D*—H⋯*A*
N1—H1⋯S1^i^	0.90 (2)	2.48 (3)	3.351 (4)	162 (2)
C13—H13*A*⋯O1^ii^	0.95	2.59	3.434 (5)	148
C18—H18*A*⋯S1^iii^	0.95	2.87	3.609 (5)	136

Symmetry codes: (i) 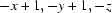; (ii) 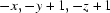; (iii) 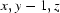.

**Fig. 1.**
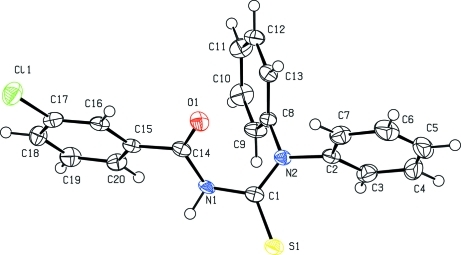
The molecular structure of (I). Displacement ellipsoids are drawn at the 50% probability level.

**Fig. 2.**
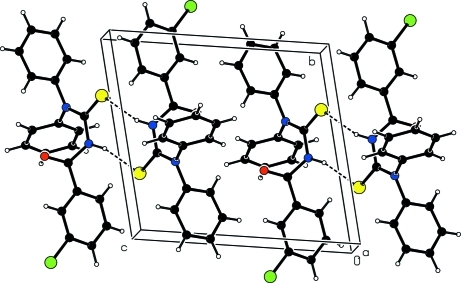
A packing diagram for (I). Hydrogen bonds are shown as dashed lines.

## supplementary crystallographic information

### Comment

Thioureas and their metal complexes are an important class of compounds with a wide range of biological applications such as antitubercular, antithroid, anthelmintic, antibacterial, insecticidal and rodenticidal properties (Schroeder, 1955; Huebhr *et al.*, 1953; Madan *et al.*, 1991). Another area of the application of thiourea derivatives is analytical chemistry where some of these compounds have been used in the liquid-liquid extraction and separation of some transition metal ions (Koch, 2001).

Recently, a number of works on the structural and spectral properties of the thiourea derivatives and their metal complexes have appeared in the literature (Özer *et al.*, 2009; Mansuroğlu *et al.*, 2008; Uğur *et al*., 2006; Arslan *et al.*, 2003c). We report here the crystal structure of one of them. The synthesis involves the reaction of a 3-chlorobenzoyl chloride with potassium thiocyanate in dry acetone followed by condensation of the 3-chlorobenzoyl isothiocyanate with the diphenylamine.

The molecular structure of the title compound, (I), is depicted in Fig. 1. The bond lengths and angles in the thiourea moiety are typical for thiourea derivatives; the C1-S1 (1.659 (3) Å) and C14-O1 (1.216 (4) Å) bonds both show typical double-bond character (Arslan *et al.*, 2003a, 2003b; Khawar Rauf *et al.* 2009, 2006; Yamin & Yusof, 2003). The C-N bond lengths C14-N1 (1.388 (4) Å), C1-N1 (1.380 (4) Å) and C1-N2 (1.336 (4) Å) are shorter than the normal C-N single-bond length of about 1.48 Å. The shortening of these C-N bonds reveals the effects of resonance in this part of the molecule (Arslan *et al.*, 2003a, 2003b; Khawar Rauf *et al.*, 2009, 2006; Yamin & Yusof, 2003). The conformation of the title molecule with respect to the thiocarbonyl and carbonyl moieties is twisted, as reflected by the C1-N1-C14-O1 and C14-N1-C1-N2 torsion angles of -4.4 (6) ^o^ and -53.3 (5) ^o^, respectively.

The atom N2 is *sp*^2^-hybridized, because of the sum of the angles around atom N2 is 359.8 (3) °. The phenyl rings are rotated out of the mean plane of the N1-C1-S1-N2 atoms by 80.1 (2) ° (C2-C7 ring) and 69.96 (19) ° (C8-C13 ring). In addition, the dihedral angle between C2-C7 ring and C8-C13 ring is 72.8 (2) °.

As can be seen from the packing diagram (Fig. 2), intermolecular N-H···S hydrogen bond (Table 1) links the molecules into dimers, which are stacked along the *c*-axis. The other intermolecular contacts, C-H···O and C-H···S, are also listed in Table 1.

### Experimental

The title compound was prepared with a procedure similar to that reported in the literature (Arslan *et al.*, 2003a). A solution of 3-chlorobenzoyl chloride (0.01 mol) in acetone (50 cm^3^) was added dropwise to a suspension of potassium thiocyanate (0.01 mol) in acetone (30 cm^3^). The reaction mixture was heated under reflux for 30 min, and then cooled to room temperature. A solution of diphenylamine (0.01 mol) in acetone (10 cm^3^) was added and the resulting mixture was stirred for 2 h. Hydrochloric acid (0.1 N, 300 cm^3^) was added to the solution, which was then filtered. The solid product was washed with water and purified by recrystalization from an ethanol:dichloromethane mixture (1:2). Anal. Calcd. for C_20_H_15_ClN_2_OS: C, 65.5; H, 4.1; N,7.6. Found: C, 65.4; H, 3.9; N, 7.7%.

### Crystal data

**Table:**

C_20_H_15_ClN_2_OS	*Z* = 2
*M**_r_* = 366.85	*F*(000) = 380
Triclinic, *P*1	*D*_x_ = 1.407 Mg m^−^^3^
Hall symbol: -P 1	Mo *K*α radiation, λ = 0.71073 Å
*a* = 8.196 (5) Å	Cell parameters from 710 reflections
*b* = 10.357 (6) Å	θ = 3.0–26.5°
*c* = 11.699 (6) Å	µ = 0.35 mm^−^^1^
α = 72.565 (10)°	*T* = 120 K
β = 70.495 (10)°	Prism, colourless
γ = 71.303 (10)°	0.49 × 0.32 × 0.10 mm
*V* = 865.8 (9) Å^3^	

### Data collection

**Table:**

Bruker SMART APEX diffractometer	3490 independent reflections
Radiation source: sealed tube	2290 reflections with *I* > 2σ(*I*)
graphite	*R*_int_ = 0.080
φ and ω scans	θ_max_ = 26.4°, θ_min_ = 2.1°
Absorption correction: multi-scan (*SADABS*; Bruker, 2002)	*h* = −10→10
*T*_min_ = 0.847, *T*_max_ = 0.966	*k* = −12→12
5401 measured reflections	*l* = −13→14

### Refinement

**Table:**

Refinement on *F*^2^	Primary atom site location: structure-invariant direct methods
Least-squares matrix: full	Secondary atom site location: difference Fourier map
*R*[*F*^2^ > 2σ(*F*^2^)] = 0.061	Hydrogen site location: difference Fourier map
*wR*(*F*^2^) = 0.146	H atoms treated by a mixture of independent and constrained refinement
*S* = 1.06	*w* = 1/[σ^2^(*F*_o_^2^) + (0.0629*P*)^2^] where *P* = (*F*_o_^2^ + 2*F*_c_^2^)/3
3490 reflections	(Δ/σ)_max_ < 0.001
230 parameters	Δρ_max_ = 0.37 e Å^−^^3^
1 restraint	Δρ_min_ = −0.31 e Å^−^^3^

### Special details

**Table:**

Geometry. All esds (except the esd in the dihedral angle between two l.s. planes) are estimated using the full covariance matrix. The cell esds are taken into account individually in the estimation of esds in distances, angles and torsion angles; correlations between esds in cell parameters are only used when they are defined by crystal symmetry. An approximate (isotropic) treatment of cell esds is used for estimating esds involving l.s. planes.
Refinement. Refinement of F^2^ against ALL reflections. The weighted R-factor wR and goodness of fit S are based on F^2^, conventional R-factors R are based on F, with F set to zero for negative F^2^. The threshold expression of F^2^ > 2sigma(F^2^) is used only for calculating R-factors(gt) etc. and is not relevant to the choice of reflections for refinement. R-factors based on F^2^ are statistically about twice as large as those based on F, and R- factors based on ALL data will be even larger.

### Fractional atomic coordinates and isotropic or equivalent isotropic displacement parameters (Å^2^)

**Table:**

	*x*	*y*	*z*	*U*_iso_*/*U*_eq_	
Cl1	0.51239 (14)	−0.17055 (10)	0.41549 (10)	0.0406 (3)	
S1	0.36004 (12)	0.69686 (9)	0.05276 (9)	0.0269 (2)	
O1	0.2248 (3)	0.3782 (2)	0.3535 (2)	0.0276 (6)	
N1	0.2744 (4)	0.4535 (3)	0.1434 (3)	0.0213 (6)	
H1	0.364 (3)	0.430 (3)	0.078 (2)	0.020 (9)*	
N2	0.0613 (3)	0.6406 (3)	0.2112 (3)	0.0210 (6)	
C1	0.2248 (4)	0.5945 (3)	0.1413 (3)	0.0210 (7)	
C2	0.0059 (4)	0.7766 (4)	0.2405 (3)	0.0226 (7)	
C3	−0.0764 (5)	0.8908 (4)	0.1661 (4)	0.0330 (9)	
H3A	−0.0918	0.8827	0.0917	0.040*	
C4	−0.1364 (5)	1.0175 (4)	0.2006 (5)	0.0464 (11)	
H4A	−0.1930	1.0975	0.1494	0.056*	
C5	−0.1154 (6)	1.0291 (5)	0.3079 (5)	0.0506 (12)	
H5A	−0.1565	1.1169	0.3309	0.061*	
C6	−0.0344 (6)	0.9132 (5)	0.3825 (4)	0.0442 (11)	
H6A	−0.0227	0.9205	0.4582	0.053*	
C7	0.0294 (5)	0.7869 (4)	0.3478 (3)	0.0322 (9)	
H7A	0.0893	0.7075	0.3977	0.039*	
C8	−0.0738 (4)	0.5643 (3)	0.2509 (3)	0.0205 (7)	
C9	−0.1246 (5)	0.5352 (4)	0.1618 (3)	0.0288 (8)	
H9A	−0.0697	0.5650	0.0763	0.035*	
C10	−0.2535 (5)	0.4636 (4)	0.1963 (4)	0.0361 (9)	
H10A	−0.2869	0.4425	0.1349	0.043*	
C11	−0.3350 (5)	0.4219 (4)	0.3204 (4)	0.0337 (9)	
H11A	−0.4245	0.3720	0.3447	0.040*	
C12	−0.2857 (5)	0.4530 (4)	0.4097 (3)	0.0291 (8)	
H12A	−0.3416	0.4243	0.4952	0.035*	
C13	−0.1559 (4)	0.5254 (4)	0.3744 (3)	0.0249 (8)	
H13A	−0.1235	0.5483	0.4353	0.030*	
C14	0.2666 (4)	0.3508 (4)	0.2519 (3)	0.0220 (7)	
C15	0.3130 (4)	0.2074 (3)	0.2337 (3)	0.0213 (7)	
C16	0.3799 (4)	0.0992 (3)	0.3218 (3)	0.0235 (8)	
H16A	0.3953	0.1180	0.3917	0.028*	
C17	0.4235 (5)	−0.0348 (4)	0.3077 (3)	0.0279 (8)	
C18	0.3970 (5)	−0.0663 (4)	0.2097 (3)	0.0299 (9)	
H18A	0.4264	−0.1604	0.2021	0.036*	
C19	0.3273 (5)	0.0413 (4)	0.1234 (3)	0.0314 (9)	
H19A	0.3074	0.0213	0.0557	0.038*	
C20	0.2864 (5)	0.1766 (4)	0.1339 (3)	0.0266 (8)	
H20A	0.2397	0.2501	0.0731	0.032*	

### Atomic displacement parameters (Å^2^)

**Table:**

	*U*^11^	*U*^22^	*U*^33^	*U*^12^	*U*^13^	*U*^23^
Cl1	0.0544 (6)	0.0299 (5)	0.0360 (6)	−0.0082 (5)	−0.0165 (5)	−0.0022 (4)
S1	0.0276 (5)	0.0304 (5)	0.0239 (5)	−0.0124 (4)	0.0013 (4)	−0.0112 (4)
O1	0.0352 (14)	0.0317 (14)	0.0189 (13)	−0.0047 (11)	−0.0097 (11)	−0.0103 (11)
N1	0.0228 (15)	0.0256 (15)	0.0138 (15)	−0.0063 (12)	0.0024 (12)	−0.0093 (12)
N2	0.0231 (15)	0.0244 (15)	0.0165 (15)	−0.0072 (12)	−0.0029 (12)	−0.0068 (12)
C1	0.0242 (17)	0.0299 (19)	0.0124 (17)	−0.0077 (15)	−0.0065 (14)	−0.0065 (14)
C2	0.0220 (17)	0.0281 (19)	0.0188 (18)	−0.0098 (15)	−0.0006 (14)	−0.0079 (15)
C3	0.033 (2)	0.034 (2)	0.028 (2)	−0.0098 (18)	−0.0074 (17)	−0.0003 (17)
C4	0.031 (2)	0.029 (2)	0.064 (3)	−0.0037 (18)	−0.003 (2)	−0.002 (2)
C5	0.050 (3)	0.039 (3)	0.060 (3)	−0.019 (2)	0.014 (2)	−0.029 (2)
C6	0.051 (3)	0.054 (3)	0.039 (3)	−0.027 (2)	0.004 (2)	−0.028 (2)
C7	0.039 (2)	0.037 (2)	0.025 (2)	−0.0152 (18)	−0.0062 (17)	−0.0088 (17)
C8	0.0216 (17)	0.0235 (17)	0.0169 (18)	−0.0060 (14)	−0.0047 (14)	−0.0048 (14)
C9	0.0303 (19)	0.043 (2)	0.0161 (18)	−0.0148 (18)	−0.0026 (15)	−0.0084 (16)
C10	0.042 (2)	0.045 (2)	0.031 (2)	−0.0138 (19)	−0.0168 (19)	−0.0099 (19)
C11	0.028 (2)	0.040 (2)	0.038 (2)	−0.0171 (18)	−0.0115 (18)	−0.0034 (18)
C12	0.0247 (19)	0.036 (2)	0.022 (2)	−0.0082 (17)	−0.0016 (16)	−0.0041 (16)
C13	0.0244 (18)	0.0297 (19)	0.0222 (19)	−0.0059 (15)	−0.0062 (15)	−0.0086 (15)
C14	0.0164 (16)	0.0313 (19)	0.0201 (19)	−0.0070 (14)	−0.0038 (14)	−0.0077 (15)
C15	0.0192 (16)	0.0293 (19)	0.0173 (18)	−0.0104 (14)	0.0000 (14)	−0.0082 (15)
C16	0.0228 (17)	0.032 (2)	0.0175 (18)	−0.0119 (15)	−0.0003 (14)	−0.0081 (15)
C17	0.0269 (18)	0.031 (2)	0.023 (2)	−0.0121 (17)	−0.0033 (15)	−0.0002 (16)
C18	0.034 (2)	0.026 (2)	0.035 (2)	−0.0126 (16)	−0.0063 (17)	−0.0135 (17)
C19	0.037 (2)	0.038 (2)	0.028 (2)	−0.0163 (18)	−0.0071 (18)	−0.0129 (18)
C20	0.0290 (19)	0.032 (2)	0.0213 (19)	−0.0114 (16)	−0.0076 (16)	−0.0045 (16)

### Geometric parameters (Å, °)

**Table:**

Cl1—C17	1.730 (4)	C8—C9	1.382 (4)
S1—C1	1.659 (3)	C9—C10	1.366 (5)
O1—C14	1.216 (4)	C9—H9A	0.9500
N1—C1	1.380 (4)	C10—C11	1.379 (5)
N1—C14	1.388 (4)	C10—H10A	0.9500
N1—H1	0.894 (10)	C11—C12	1.387 (5)
N2—C1	1.336 (4)	C11—H11A	0.9500
N2—C8	1.437 (4)	C12—C13	1.378 (5)
N2—C2	1.442 (4)	C12—H12A	0.9500
C2—C3	1.369 (5)	C13—H13A	0.9500
C2—C7	1.372 (5)	C14—C15	1.473 (5)
C3—C4	1.378 (5)	C15—C16	1.382 (5)
C3—H3A	0.9500	C15—C20	1.395 (4)
C4—C5	1.367 (6)	C16—C17	1.364 (5)
C4—H4A	0.9500	C16—H16A	0.9500
C5—C6	1.375 (6)	C17—C18	1.380 (5)
C5—H5A	0.9500	C18—C19	1.375 (5)
C6—C7	1.374 (5)	C18—H18A	0.9500
C6—H6A	0.9500	C19—C20	1.367 (5)
C7—H7A	0.9500	C19—H19A	0.9500
C8—C13	1.370 (5)	C20—H20A	0.9500
			
C1—N1—C14	123.6 (3)	C9—C10—C11	119.9 (3)
C1—N1—H1	114 (2)	C9—C10—H10A	120.0
C14—N1—H1	115 (2)	C11—C10—H10A	120.0
C1—N2—C8	122.0 (3)	C10—C11—C12	119.8 (3)
C1—N2—C2	121.4 (3)	C10—C11—H11A	120.1
C8—N2—C2	116.3 (3)	C12—C11—H11A	120.1
N2—C1—N1	115.3 (3)	C13—C12—C11	120.1 (3)
N2—C1—S1	123.8 (3)	C13—C12—H12A	120.0
N1—C1—S1	120.9 (3)	C11—C12—H12A	120.0
C3—C2—C7	121.0 (3)	C8—C13—C12	119.7 (3)
C3—C2—N2	120.6 (3)	C8—C13—H13A	120.2
C7—C2—N2	118.3 (3)	C12—C13—H13A	120.2
C2—C3—C4	119.0 (4)	O1—C14—N1	122.1 (3)
C2—C3—H3A	120.5	O1—C14—C15	123.2 (3)
C4—C3—H3A	120.5	N1—C14—C15	114.7 (3)
C5—C4—C3	120.6 (4)	C16—C15—C20	119.1 (3)
C5—C4—H4A	119.7	C16—C15—C14	118.1 (3)
C3—C4—H4A	119.7	C20—C15—C14	122.7 (3)
C4—C5—C6	119.8 (4)	C17—C16—C15	119.5 (3)
C4—C5—H5A	120.1	C17—C16—H16A	120.3
C6—C5—H5A	120.1	C15—C16—H16A	120.3
C7—C6—C5	120.2 (4)	C16—C17—C18	121.8 (3)
C7—C6—H6A	119.9	C16—C17—Cl1	119.8 (3)
C5—C6—H6A	119.9	C18—C17—Cl1	118.4 (3)
C2—C7—C6	119.3 (4)	C19—C18—C17	118.6 (3)
C2—C7—H7A	120.3	C19—C18—H18A	120.7
C6—C7—H7A	120.3	C17—C18—H18A	120.7
C13—C8—C9	120.3 (3)	C20—C19—C18	120.7 (3)
C13—C8—N2	120.9 (3)	C20—C19—H19A	119.7
C9—C8—N2	118.7 (3)	C18—C19—H19A	119.7
C10—C9—C8	120.2 (3)	C19—C20—C15	120.3 (3)
C10—C9—H9A	119.9	C19—C20—H20A	119.9
C8—C9—H9A	119.9	C15—C20—H20A	119.9
			
C8—N2—C1—N1	−20.3 (4)	N2—C8—C9—C10	−179.6 (3)
C2—N2—C1—N1	166.0 (3)	C8—C9—C10—C11	0.9 (6)
C8—N2—C1—S1	157.6 (2)	C9—C10—C11—C12	0.1 (6)
C2—N2—C1—S1	−16.2 (4)	C10—C11—C12—C13	0.0 (6)
C14—N1—C1—N2	−53.3 (4)	C9—C8—C13—C12	2.1 (5)
C14—N1—C1—S1	128.9 (3)	N2—C8—C13—C12	179.6 (3)
C1—N2—C2—C3	91.3 (4)	C11—C12—C13—C8	−1.1 (5)
C8—N2—C2—C3	−82.8 (4)	C1—N1—C14—O1	−4.4 (5)
C1—N2—C2—C7	−92.0 (4)	C1—N1—C14—C15	175.7 (3)
C8—N2—C2—C7	93.9 (4)	O1—C14—C15—C16	−26.1 (5)
C7—C2—C3—C4	−0.2 (5)	N1—C14—C15—C16	153.8 (3)
N2—C2—C3—C4	176.4 (3)	O1—C14—C15—C20	151.6 (3)
C2—C3—C4—C5	−0.4 (6)	N1—C14—C15—C20	−28.4 (4)
C3—C4—C5—C6	−0.4 (6)	C20—C15—C16—C17	2.0 (5)
C4—C5—C6—C7	1.8 (6)	C14—C15—C16—C17	179.8 (3)
C3—C2—C7—C6	1.5 (5)	C15—C16—C17—C18	−2.3 (5)
N2—C2—C7—C6	−175.1 (3)	C15—C16—C17—Cl1	178.4 (2)
C5—C6—C7—C2	−2.3 (6)	C16—C17—C18—C19	1.1 (5)
C1—N2—C8—C13	123.8 (4)	Cl1—C17—C18—C19	−179.7 (3)
C2—N2—C8—C13	−62.2 (4)	C17—C18—C19—C20	0.5 (5)
C1—N2—C8—C9	−58.6 (4)	C18—C19—C20—C15	−0.8 (5)
C2—N2—C8—C9	115.4 (4)	C16—C15—C20—C19	−0.5 (5)
C13—C8—C9—C10	−2.0 (5)	C14—C15—C20—C19	−178.2 (3)

### Hydrogen-bond geometry (Å, °)

**Table:**

*D*—H···*A*	*D*—H	H···*A*	*D*···*A*	*D*—H···*A*
N1—H1···S1^i^	0.90 (2)	2.48 (3)	3.351 (4)	162 (2)
C13—H13A···O1^ii^	0.95	2.59	3.434 (5)	148
C18—H18A···S1^iii^	0.95	2.87	3.609 (5)	136

Symmetry codes: (i) −*x*+1, −*y*+1, −*z*; (ii) −*x*, −*y*+1, −*z*+1; (iii) *x*, *y*−1, *z*.
